# An assessment of the effect of hepatitis B vaccine in decreasing the amount of hepatitis B disease in Italy

**DOI:** 10.1186/1743-422X-5-84

**Published:** 2008-07-24

**Authors:** Giuseppe La Torre, Nicola Nicolotti, Chiara de Waure, Giacomina Chiaradia, Maria Lucia Specchia, Alice Mannocci, Walter Ricciardi

**Affiliations:** 1 Catholic University of the Sacred Heart, Institute of Hygiene, Rome, Italy

## Abstract

**Background:**

Hepatitis B (HBV) infection is an important cause of morbidity and mortality and it is associated to a higher risk of chronic evolution in infected children. In Italy the anti-HBV vaccination was introduced in 1991 for newborn and twelve years old children. Our study aims to evaluate time trends of HBV incidence rates in order to provide an assessment of compulsory vaccination health impact.

**Method:**

Data concerning HBV incidence rates coming from Acute Viral Hepatitis Integrated Epidemiological System (SEIEVA) were collected from 1985 to 2006. SEIEVA is the Italian surveillance national system that registers acute hepatitis cases. Time trends were analysed by joinpoint regression using Joinpoint Regression Program 3.3.1 according to Kim's method. A joinpoint represents the time point when a significant trend change is detected. Time changes are expressed in terms of the Expected Annual Percent Change (EAPC) with 95% confidence interval (95% CI).

**Results:**

The joinpoint analysis showed statistically significant decreasing trends in all age groups. For the age group 0–14 EAPC was -39.0 (95% CI: -59.3; -8.4), in the period up to 1987, and -12.6 (95% CI: -16.0; -9.2) thereafter. EAPCs were -17.9 (95% CI: -18.7; -17.1) and -6.7 (95% CI: -8.0; -5.4) for 15–24 and ≥25 age groups, respectively. Nevertheless no joinpoints were found for age groups 15–24 and ≥25, whereas a joinpoint at year 1987, before compulsory vaccination, was highlighted in 0–14 age group. No joinpoint was observed after 1991.

**Discussion:**

Our results suggest that the introduction of compulsory vaccination could have contribute partly in decreasing HBV incidence rates. Compulsory vaccination health impact should be better investigated in future studies to evaluate the need for changes in current vaccination strategy.

## Background

HBV infection is an important cause of morbidity and mortality. The World Health Organisation (WHO) estimates that two billion of people worldwide have a serological evidence of past or present HBV infection [1].

The prevalence of chronic HBV infection is low (<2%) in the general population in Northern and Western Europe, North America, Australia, New Zealand, Mexico, and Southern South America. The prevalence of chronic HBV infection is intermediate (2%–7%) in South Central and Southwest Asia, Israel, Japan, Eastern and Southern Europe, Russia, most areas surrounding the Amazon River basin, Honduras, and Guatemala. The prevalence of chronic HBV infection is high (>8%) in all Countries in Africa, Southeast Asia, the Middle East (except Israel), Southern and Western Pacific islands, the interior Amazon River basin and certain parts of the Caribbean (Haiti and the Dominican Republic) [2].

In Italy, the prevalence of HBV infection is set under 2% from the beginning of the twentieth. The most important routes of transmission are sexual intercourse, intrafamiliar contacts and i.v. drug use [3]. The HBV infection trend is changed through the years. There were two important downward tendencies in the serum prevalence of infection, one at the beginning of the eighties, related to the improved socio-economic conditions and to the reduction in family numerousness [4], and one at the end of the eighties, after the spreading of HIV infection and before compulsory vaccination.

In 1985, the Acute Viral Hepatitis Integrated Epidemiologic System data (SEIEVA) was established [5]. The national surveillance system underlined an impressive reduction of the incidence of HBV infection from 12/100,000 to 5.1/100,000 through the 1985–1991 period, reporting the highest number of cases among individuals 15–24 years old and among males [6]. From the starting of compulsory vaccination campaign, in 1991, there was another downfall in HBV incidence with a reduction of 40% from 1988–91 to 1991–99. The incidence reduction was of 66% among 0–14 years old individuals and 59% among 15–24 years old ones [6].

The compulsory vaccination was mainly introduced by the high risk of chronic evolution of the infection in children. SEIEVA data demonstrated a stabilisation of the epidemiological trend of infection with a mean incidence of 1.65 cases for 100,000 in the last 6 years available [7]; this trend was also demonstrated in other European nations [8].

Anti-hepatitis B vaccine has still some aspects, such as the immunity memory length and the failure rate, to go in deep [9,10].

Our study aims to evaluate the epidemiology of HBV infection in Italy and to provide an assessment of compulsory vaccination health impact by studying time trends through the use of the joinpoint regression. This statistical technique highlights the time points that divide periods characterised by different time trends. It should so represent an innovative approach to in depth investigate the HBV incidence rates decreasing trends described by other authors [6,8,11-14] and to give some additional insight to the vaccine impact.

## Methods

### Data and setting

HBV incidence rates, reported by the SEIEVA, were collected from 1985 to 2006 [7]. SEIEVA is a surveillance system that covers 57% of Italian population and aims to investigate epidemiology of viral acute hepatitis. The system is coordinated by the National Institute of Health and each Local Health Unit (LHU) can join voluntary the system.

Data regarded incidence rates for 100,000 and were stratified by age (0–14; 15–24; ≥ 25). Rates were computed dividing the number of cases by the total population of each joining LHU. In the surveillance system the diagnosis of acute hepatitis B was posed if a serologically confirmed positivity for IgM anti-HBcAg was found.

Since SEIEVA data were available before mass vaccination introduction for the period 1985–1991, study of time trend changes was made possible.

In Italy another national database on HBV infections (SIMI) exists from Italian Public Health Ministry [15]. However, this database is not exclusively devoted to this type of infection, but covers all notifiable infectious diseases. We were not allowed to perform the same evaluation, done with SEIEVA surveillance, since SIMI data were available from 1996 only.

### Statistical analysis

The analysis on SEIEVA data was carried out for three different age groups (0–14; 15–24; over 25 years) and for all ages together. Incidence rates time trends were analysed by joinpoint regression according to Kim's method [16].

The following formula was used for the logarithmic transformation of incidence rates:

*ln(y) *= *bx*

where x represents the calendar years, b is the regression coefficient and y the incidence rate.

A joinpoint represents the time point when a significant trend change is detected. Time changes are expressed in terms of Expected Annual Percent Change (EAPC) with respective 95% confidence interval; significance level of time trends is also reported. The null hypothesis was tested using a maximum of three changes in slope with an overall significance level of 0.05 divided by the number of join-points in the final model.

For the analysis we used the Joinpoint Regression Program, Version 3.3.1 [17].

## Results

**Figure 1:**
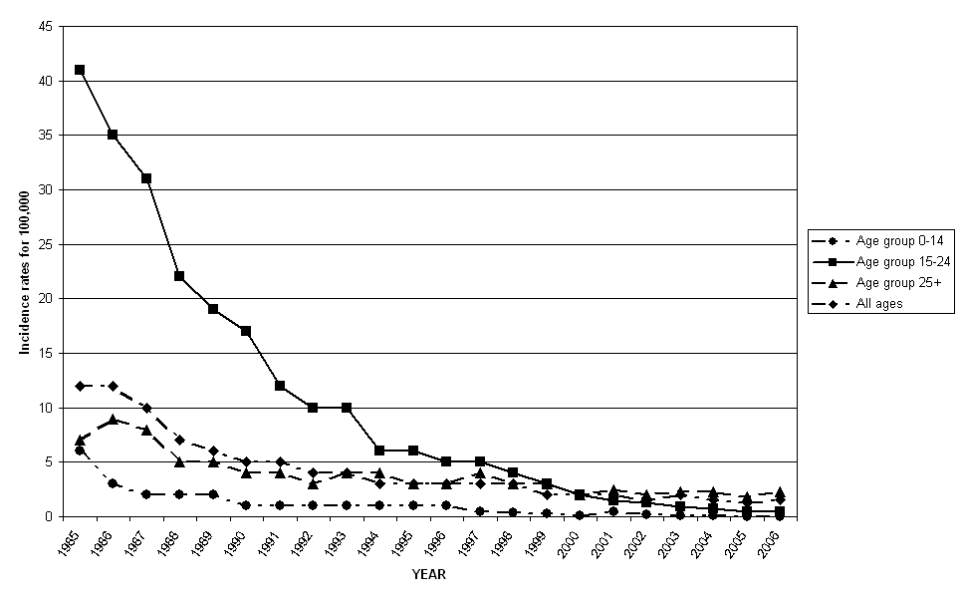
Incidence rates (for 100,000) of HBV infection in Italy, 1985–2006.

In the 1985–2006 period a strong reduction of hepatitis B incidence rates in all age groups was observed (Figure 1).

SEIEVA data showed the highest incidence rates of hepatitis B in individuals belonging to the 15–24 and ≥25 age groups. The incidence rate reduction goes from 6.00 to 0.02 for 100,000 in the age class 0–14, from 41.00 to 0.50 in the group 15–24 years and from 7.00 to 2.30 in individuals of 25 years or more, in the period 1985–2006. Considering all the age groups, the incidence rate decreased from 12 for 100,000 to 1.6 for 100,000.

The joinpoint analysis showed a statistically significant decrease of HBV infection incidence rates too, in particular in 0–14 and 15–24 age groups.

For the age group 0–14 the analysis highlighted a joinpoint at year 1987; EAPC changed from -39.0 (95% CI: -59.3; -8.4), in the period up to 1987, to -12.6 (95% CI: -16.0; -9.2) thereafter thus meaning that from 1987 HBV incidence rates showed a significant overall annual decrease of 12.6% (Table 1).

**Table 1:** EAPC and 95% CI

**Age group**	**Years range**	**EAPC (%)**	**95% CI**	**p-value**
**0–14**	1985–1987	-39.0	(-59.3; -8.4)	0.02
	1987–2006	-12.6	(-16.0; -9.2)	<0.001
				
**15–24**	1985–2006	-17.9	(-18.7; -17.1)	<0.001
				
**Over 25**	1985–2006	-6.7	(-8.0; -5.4)	<0.001
				
**All**	1985–1992	-15.6	(-18.4; -12.8)	<0.001
	1992–2006	-7.1	(-9.0; -5.1)	<0.001

No joinpoints were found for the other age groups. EAPCs were -17.9 (95% CI: -18.7; -17.1) and -6.7 (95% CI: -8.0; -5.4) for 15–24 and ≥25 age groups: HBV incidence rates showed an annual decrease of 17.6% and 6.7% respectively (Table 1).

On the other hand, considering all age groups a joinpoint at year 1992 was detected; overall annual decrease was of 15.6% (95% CI: -18.4; -12.8) before 1992 and 7.1% (95% CI: -9.0; -5.1) thereafter (Table 1).

**Figure 2:**
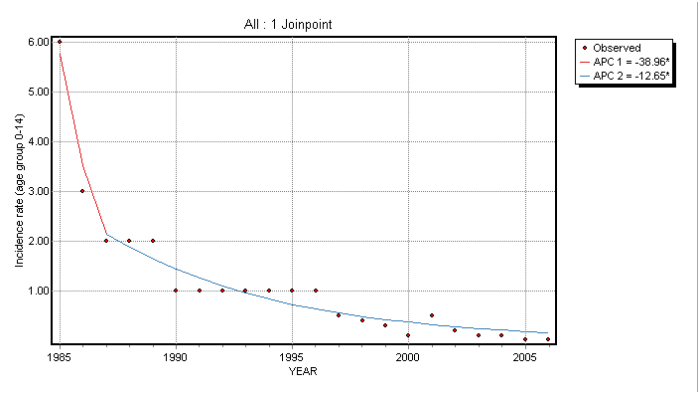
Joinpoint regression for 0–14 age group.

**Figure 3:**
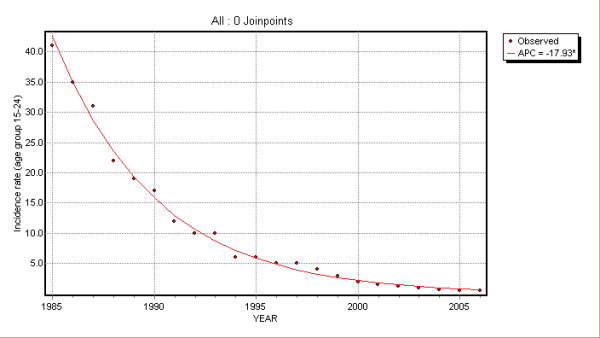
Joinpoint regression for 15–24 age group.

**Figure 4:**
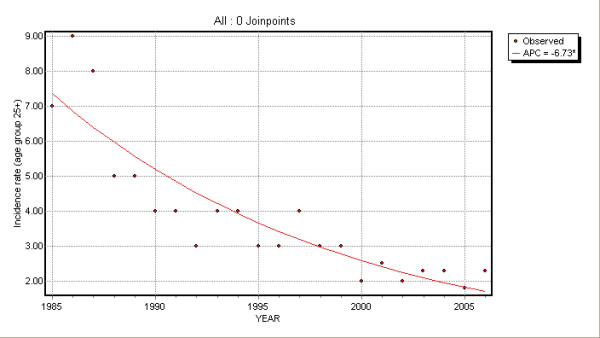
Joinpoint regression for 25+ age group.

**Figure 5:**
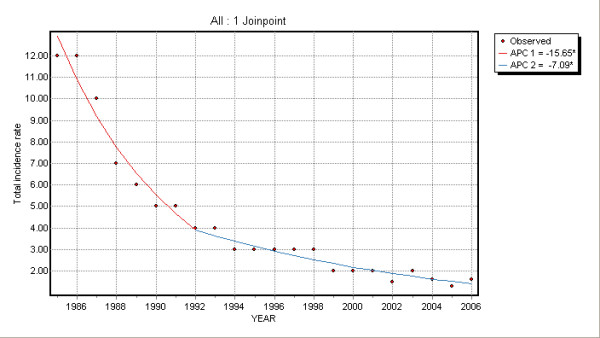
Joinpoint regression for all ages.

Time trend changes are illustrated in Figures 2, 3, 4 and 5.

## Discussion

Hepatitis B incidence rates decreased in each age group throughout the period considered.

From the analysis of time trends, it is possible to suppose that the reduction of HBV incidence rates was influenced not only by mass vaccination. Moreover, considering that vaccination coverage reached about 95% since 1991 [18], other factors (i.e. different lifestyles, new hygiene rules and the introduction of different systems of prevention such as the blood screening and the use of precautions in medical setting), besides vaccination campaign, could have contributed to the decrease of HBV infections.

According to the joinpoint analysis of SEIEVA data, a statistically significant change in HBV incidence rates time trend was found, before the introduction of compulsory vaccination, for the age group 0–14 (up to 1087). In particular, a smaller decrease of HBV incidence rates in the following period (1987–2005) than in the first one (1985–1987) was observed. Nevertheless, in this age group, prevalence rates of HBV serological markers were estimated to be low in low/intermediate endemic areas for the infection [19]. Moreover, the decrease of HBV incidence rates before compulsory vaccination could be related to the strongly recommendation of HBsAg screening for pregnant women during the last trimester of pregnancy since 1984 [20]. In low/intermediate endemic areas, such as Italy as a whole, and such as the other Southern Mediterranean European regions, horizontal transmission is the main way of acquiring infection thus determining the highest HBV incidence rates among adults [21]. Improved sanitation, obtained with the use of universal precautions in medical settings and blood screening, social, behavioural and demographic changes and sexual educational campaigns seem yet to have been effective to reduce horizontal transmission in these countries and there are some evidences that the highest HBV incidence rates have to be expected in adults older than 50 [19,22]. These same changes could be positively associated to the decrease of HBV incidence rates observed among people from 15 to 24 years of age and in 25 years or older people. HBV incidence rates have progressively decreased through the years in all age groups, even if EAPC was smaller in over 25 years old than in the other groups. The incidence rate reduction in over 25 years people could be also partly attributed to the herd immunity induced by the high coverage rate of children immunisations [23].

The introduction of compulsory vaccination has determined a reduction of HBV incidence rates and this decrease, according to our analysis, could have been influenced not only by primary prevention sustained by vaccination stategies. This could be also sustained from the evidence of a joinpoint at the year 1992. After this year there was a smaller decrease in HBV incidence rates than before. Moreover, the vaccination of high risk adults, such as injection drugs users and persons at risk of sexual transmission, should be promoted. In fact, there are evidences that these groups of adults, despite of recommendations, are not used to be vaccinated [24,11,12]. This is also confirmed by EAPC value.

Our study has some strenght and limitations. As far as concerns the former ones, this is the first time that the Joinpoint regression model was used in assessing the time trends of a particular infectious in Italy, thus allowing to give some insight to effectiveness of a specific vaccination campaign. The principal limit of our study was concerning the internal validity: unfortunately there is a lack of data before 1985, that could have helped us to better estimate time trend changes in HBV incidence rates. Finally, another problem is related to external validity: the use of SEIEVA data system that could not completely represent the national epidemiological setting. Nevertheless, it should be considered that the wide distribution of LHUs allows standard approaches and procedures to anti-HBV vaccination [13].

This study still underlines the importance of a correct and exhaustive data collection in a surveillance system to realise survey on efficacy of public health interventions.

Since the results of this study could be considered preliminary, we would suggest to carry out new evidences about anti-HBV vaccine health impact to evaluate the possible need to modify current vaccination strategy.

## Authors' contributions

GLT designed the study and guided the statistical analysis. NN and CdW collected data and performed the statistical analysis. GC and MLS drafted the manuscript. AM verified the results. WR coordinated the working group and reviewed the paper. All authors read and approved the final manuscript.
